# Rapunzel Syndrome Requiring Open Surgical Excision in an Adolescent Female With Autism Spectrum Disorder: A Case Report

**DOI:** 10.7759/cureus.111103

**Published:** 2026-06-18

**Authors:** David A Febre-Alemañy, Juan C Bonilla-Robles, Miguel Serpa-Irizarry, Juan Berríos-Cordero, Jennyvette Trinidad-Pineiro, Anwar Abdul-Hadi

**Affiliations:** 1 Department of Surgery, San Juan Bautista School of Medicine, Caguas, PRI; 2 Department of Surgery, University of Puerto Rico, Medical Sciences Campus, San Juan, PRI; 3 Department of Surgery, University of Puerto Rico School of Medicine, San Juan, USA; 4 Department of Surgery, Division of Pediatric Surgery, University of Puerto Rico, Medical Sciences Campus, San Juan, PRI

**Keywords:** autism spectrum disorder (asd), gastric trichobezoar, laparotomy for bezoar, pediatric, rapunzel syndrome

## Abstract

Trichobezoar is a rare but clinically significant cause of gastrointestinal obstruction, typically affecting adolescent females with underlying psychiatric or neurodevelopmental conditions. We report an unusual case of synchronous gastric and duodenal trichobezoar in a 17-year-old female with autism spectrum disorder (ASD) whose guardian denied trichotillomania or pica, highlighting the diagnostic and surgical challenges this presentation poses. The patient presented with a three-week history of diffuse abdominal pain, nausea, and bilious non-bloody emesis. Abdominopelvic CT with contrast demonstrated a continuous large mass in the stomach and duodenum, interpreted as bezoars. Pain was rated 6/10 at presentation, predominantly in the upper quadrants. Diagnostic endoscopy confirmed a trichobezoar at the proximal stomach blocking passage into the pylorus. The patient underwent open exploratory laparotomy with gastrotomy for excision of the gastric trichobezoar and proximal jejunum enterotomy for excision of a second trichobezoar drained from the distal duodenum. The bowel and stomach were closed with running PDS sutures and Lembert reinforcement. Estimated blood loss was 5 cc. The patient was extubated and transferred to the pediatric acute care unit (PACU) in stable condition.

This report underscores the importance of considering trichobezoar in adolescent females with neurodevelopmental disorders presenting with upper gastrointestinal symptoms, even in the absence of a reported history of hair ingestion. Multidisciplinary surgical and psychiatric follow-up is essential to prevent recurrence.

## Introduction

Bezoars are uncommon but clinically important causes of gastrointestinal obstruction, and their management is guided by bezoar size, location, composition, obstructive burden, and feasibility of safe extraction. The most extreme form, Rapunzel syndrome, occurs when the trichobezoar extends from the stomach into the duodenum [1]. Trichobezoars are associated with significant morbidity due to their insidious onset and the often delayed recognition of the underlying behavior [2]. Presenting symptoms of abdominal pain, nausea, vomiting, and early satiety are nonspecific. Patients with neurodevelopmental, psychiatric, or cognitive conditions may face additional barriers to timely diagnosis and treatment, including variable symptom expression, differences in expressive communication, repetitive or compulsive behaviors, or limited awareness of the medical significance of the behavior. These factors should be evaluated on an individualized basis, with consideration of each patient’s baseline cognitive and communicative abilities [3].

Although endoscopic or minimally invasive approaches may be feasible in selected patients, large obstructing bezoars, Rapunzel syndrome, complete mechanical obstruction, or extension beyond the stomach often require laparotomy and removal. Published literature includes a retrospective study of 45 patients with gastrointestinal bezoars in which 86.7% required surgical intervention, postoperative complications occurred in 41%, and laparoscopy, although attempted in approximately half of surgical cases, required conversion to an open procedure in 55% of attempts [4].

These findings support that minimally invasive management is not universally applicable and may be limited by bezoar burden, prior abdominal surgery, location, bowel distension, adhesions, and technical exposure. Laparoscopy is safe and effective in bezoar-induced small-bowel obstruction, with outcomes dependent on surgeon expertise [5]. Laparoscopic removal yields shorter operative times, fewer postoperative complications, and shorter hospital stays compared with open surgery; however, conversion remains necessary in selected cases because of technical difficulty, inability to crush the bezoar, or inability to advance the obstructing mass safely [6]. Therefore, the operative approach should be individualized rather than presented as a simple open-versus-minimally invasive choice.

In gastric bezoars, endoscopic and chemical dissolution are first-line therapies. Drug lithotripsy with sodium bicarbonate and a proton pump inhibitor has achieved 85% clearance within 48 hours and 98% within one week. When trichobezoars are localized to the small bowel, surgical exploration and milking the mass distally into the cecum is preferred; if this is unsuccessful, enterotomy represents the next best approach [7]. In Rapunzel syndrome, laparotomy remains the gold standard because of the transpyloric position of these masses. Laparoscopic removal through a Pfannenstiel incision has been described, offering cosmetic advantages in pediatric patients [7]. This approach is not feasible if postpyloric extension of the mass is present, as it is in 71% of cases. Novel guidewire-based tangential sawing fragmentation (GTSF) has achieved a 100% success rate in giant bezoars (>5 cm), with an average fragmentation time of only eight minutes and without complications [8].

In patients with neurodevelopmental or psychiatric comorbidities, definitive management also requires recurrence prevention through psychiatric evaluation, behavioral intervention, caregiver education, and longitudinal follow-up, because surgical extraction alone does not address the underlying behavior driving hair ingestion. The preferred nonpharmacologic treatment for these patients is habit reversal therapy (HRT) and acceptance-enhanced behavioral therapy (AEBT) to treat the underlying trichotillomania and/or trichophagia. HRT has demonstrated superior evidence-based effect sizes (SMD = -1.22) versus controls [7,8]. Pharmacologic therapy with NAD, aripiprazole, and monoamine oxidase inhibitors has shown success in treating patients with trichotillomania, although these are deemed off-label treatment strategies [9].

We report the case of a 17-year-old female patient with autism spectrum disorder (ASD) and a three-week history of progressive abdominal pain and emesis who was found to have synchronous gastric and duodenal trichobezoars requiring open surgical excision, despite caretakers’ denial of observing trichotillomania or hair ingestion.

## Case presentation

The patient was a 17-year-old female with a past medical history significant for bronchial asthma, attention-deficit hyperactivity disorder (ADHD), and ASD. Approximately three weeks before transfer, the patient developed diffuse abdominal pain accompanied by nausea. Initial evaluation at the University Hospital (Hospital Universitario Dr. Ramon Ruiz Arnau (HURRA)) resulted in discharge with gabapentin and famotidine. Abdominal pain persisted intermittently and acutely worsened four days before transfer, followed by persistent bilious, non-bloody emesis. The day before transfer, markedly decreased oral intake prompted a repeat visit to HURRA, where the patient received intravenous fluids and pain control before discharge with return precautions.

On the day of transfer, due to escalating pain, the patient again presented to HURRA. After analgesia with ketorolac, an abdominopelvic CT scan with contrast demonstrated marked distention of the stomach and proximal duodenum with a heterogeneous intraluminal pseudomass within the gastric lumen compatible with a possible bezoar, as well as diffuse inflammatory gastric wall thickening predominant at the antropyloric region (Figures 1A, 1B, 1C).

**Figure 1:**
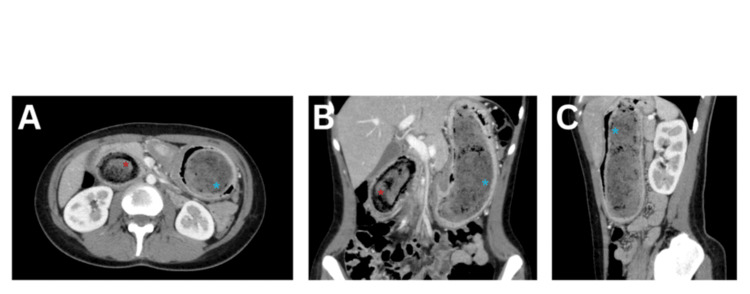
CT images of the trichobezoar (A) Transverse, (B) coronal, and (C) sagittal views. Blue asterisk: gastric component of the trichobezoar. Red asterisk: duodenal component of the trichobezoar CT: computed tomography

No inflammatory changes were identified in the right iliac fossa. The remaining solid organs and bowel were unremarkable. The patient was subsequently transferred to the Pediatric University Hospital Dr. Antonio Ortíz (Hospital Pediátrico Universitario Dr. Antonio Ortíz (HOPU)) for further management. At presentation, pain was rated 6/10, diffuse but more pronounced in the upper quadrants without radiation. She denied fever, chills, or other systemic symptoms. She also denied any history of trichotillomania, pica, hair ingestion, foreign body ingestion, and recent changes in diet or weight. She was on no current medications, and her immunizations were age-appropriate. There was no known family history of gastrointestinal malignancy, inflammatory bowel disease, or psychiatric disorders. Social history was notable for the neurodevelopmental diagnoses previously mentioned; no substance use was reported. The patient attended school and did not receive behavioral therapy. Family and psychosocial history were otherwise noncontributory.

At HOPU, abdominal examination revealed a soft but distended abdomen with diffuse tenderness, most marked in the epigastric and periumbilical regions. A firm, ill-defined mass was palpable in the epigastric area. Bowel sounds were present. There was no guarding or rigidity. The patient’s temperature was 37 °C, blood pressure was 117/69 mmHg, heart rate was 87 beats per minute (bpm), and respiratory rate was 20 breaths per minute.

Laboratory evaluation at presentation revealed a low serum bicarbonate (20 mEq/L) with an elevated anion gap (20 mEq/L), elevated urine ketones (≥80 mg/dL), and a low-normal serum glucose (68 mg/dL), collectively consistent with a starvation ketoacidosis pattern in the setting of poor oral intake and reported prolonged emesis. Nutritional status appeared compromised, as evidenced by the ketonuria and borderline BUN/creatinine ratio (20.0), suggesting catabolism and mild volume depletion secondary to inadequate caloric intake and ongoing gastrointestinal losses. Prealbumin and albumin levels were within normal limits.

Management

After evaluating the clinical and imaging findings, there was a high suspicion of a trichobezoar; hence, it was decided to manage accordingly. Informed consent was obtained, and the patient was taken to the operating room for diagnostic endoscopy, which was performed with a pediatric endoscope. A trichobezoar was identified at the proximal stomach, confirming the diagnosis. Given the size and location of the bezoar, non-operative interventions were not warranted, and the decision was made to proceed with open surgical excision.

A midline supra-umbilical incision was performed. The stomach was identified and found to be markedly distended with a hard mass palpable on external examination. A gastrotomy was performed on the anterior surface of the stomach. A continuous bolus of trichobezoar that spanned the greater curvature of the stomach and crossed the pyloric sphincter into the proximal duodenum was identified, confirming a case of Rapunzel syndrome. This mass was separated into two portions. The larger portion (Figure 2, green asterisk) was removed through the gastrostomy. The smaller portion (Figure 2, orange asterisk), located in the distal jejunum, was milked distally into the proximal jejunum, where a transverse enterotomy was performed for removal. An intraoperative image of trichobezoar extraction is shown in Figure 2. The enterotomy was closed with a running full-thickness PDS 3-0 suture followed by Lembert sutures using PDS, with a patent lumen confirmed after approximation. Closure of the gastrostomy followed the same principles described above.

**Figure 2:**
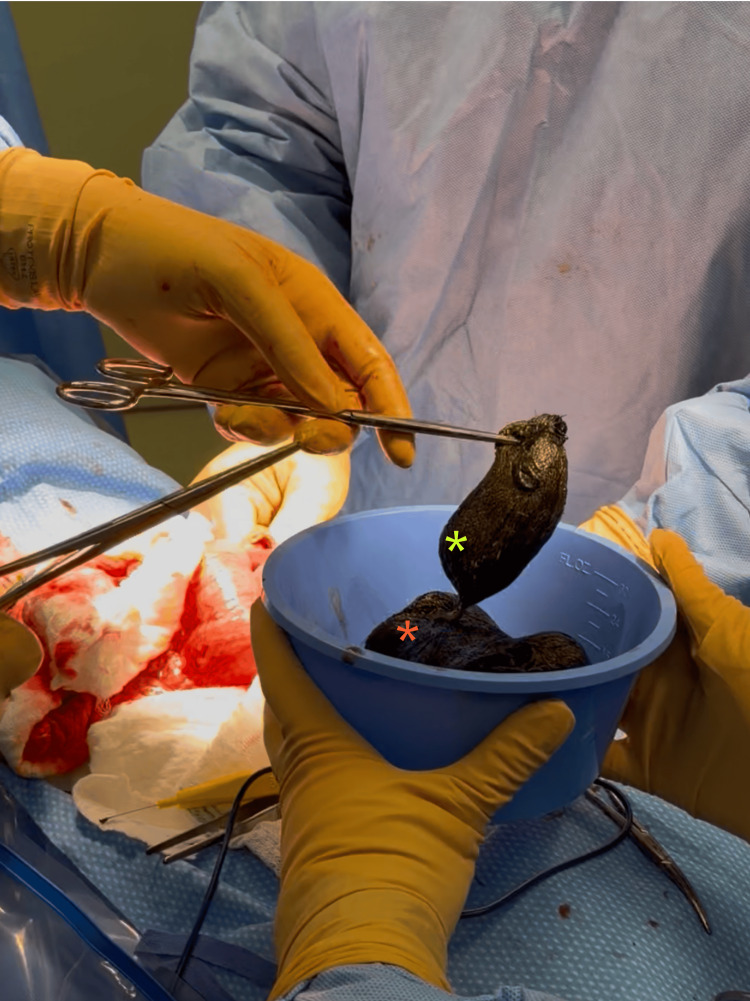
Intraoperative image of the extracted trichobezoar Duodenal portion of trichobezoar (green asterisk) measuring 8.5 cm in length and 2 cm in diameter (distal is closest to the instrument). Gastric portion of trichobezoar (orange asterisk) measuring 15.5 cm in length and 4.5 cm in diameter

The abdominal cavity was irrigated with Irrisept solution and suctioned. The abdomen was closed in layers: fascia was approximated with PDS 1-0 and skin with Monocryl 3-0. Dermabond was applied to the skin. There were no intraoperative complications. Estimated blood loss was 5 cc. The patient was extubated at the end of the procedure, and the Foley catheter was removed. She was transferred to the pediatric acute care unit (PACU) in stable condition.

Outcomes and follow-up

The postoperative course was unremarkable. On postoperative day one, the patient was hemodynamically stable, in no acute distress, and ambulating independently. Her abdomen was soft and nondistended on examination, and the midline incision was intact, exhibiting only mild periincisional tenderness without evidence of herniation, dehiscence, seroma, or infection, which was adequately controlled with ketorolac. She was initiated on a clear liquid diet and advanced stepwise over the following days. A full liquid diet was initiated on postoperative day two, a blenderized diet on postoperative day three, and a regular diet by postoperative day four, with each transition tolerated without nausea or emesis. Return of bowel function was documented on postoperative day two. Intravenous antibiotics were administered for a five-day course.

Before discharge, a psychiatric consultation was obtained, given the behavioral etiology and high recurrence rate of trichobezoars. The consulting psychiatrist identified a significant history of body-focused repetitive behaviors beginning in early childhood, including recurrent trichophagia, as well as ingestion of nails, erasers, and paper. A history of hair-pulling with subsequent ingestion around age 13, reported by the patient's mother, was noted as consistent with trichotillomania-associated trichophagia. At the time of evaluation, the patient’s guardian denied depressive symptoms, psychosis, and suicidal or homicidal ideation in the patient. The patient and her mother were counseled on the behavioral contributors to trichobezoar formation and the importance of outpatient psychiatric follow-up. A plan was established for the continuation of psychotherapy and initiation of outpatient evaluation at the University of Puerto Rico Psychiatry clinics, with which the mother agreed.

## Discussion

We discussed a case of synchronous gastric and duodenal trichobezoar, consistent with Rapunzel syndrome, in a 17-year-old female with ASD who required open surgical excision. This case is notable for the presence of a gastric bezoar that extended into the duodenum in a patient with a diagnosis of ASD and no known history of trichotillomania. Trichobezoars occur almost exclusively in young females and are strongly associated with psychiatric and neurodevelopmental conditions, most commonly trichotillomania and trichophagia [2]. The association with ASD is less well characterized in the literature, but is consistent with the known overlap between ASD and repetitive, compulsive behaviors, including pica [3,10]. Patients, particularly those with communication or behavioral challenges, may not disclose or may genuinely not recognize hair ingestion as a behavior, underscoring the need for clinical suspicion independent of the reported history.

The diagnosis of trichobezoar is best established with CT imaging, which typically demonstrates a heterogeneous, mottled intraluminal mass with air trapped within its interstices. Endoscopy provides definitive characterization and allows for assessment of mucosal injury [5]. In cases of Rapunzel syndrome (like the one presented) where the bezoar is too large for endoscopic removal, open surgery remains the standard of care, though laparoscopic approaches have been described [11,12].

The management of trichobezoars is strongly dependent on size. Small trichobezoars are preferentially treated by endoscopic removal [8]. Other cases are removed via laparoscopic-assisted minilaparotomy (LAML), combining laparoscopic visualization with a small incision (mostly Pfannenstiel) for specimen extraction. Although this approach has been proven efficient in pediatric patients [9], the senior author opted for an open approach due to the size and anatomical location of the mass in the present case.

The Rapunzel syndrome variant, in which a gastric trichobezoar extends a tail into the duodenum, is associated with higher morbidity, including obstruction, ulceration, perforation, and intussusception [2]. Our patient's presentation was consistent with the described syndrome, as the bezoar spans the greater curvature of the stomach, pyloric sphincter, and proximal duodenum. Milking the distal bezoar proximally into the jejunum for a controlled enterotomy, rather than attempting distal retrieval, is a sound operative strategy that minimizes the risk of duodenal injury.

## Conclusions

This report highlights the importance of maintaining a high index of suspicion for trichobezoar in adolescent females with neurodevelopmental disorders presenting with upper gastrointestinal symptoms, even when the patient denies a history of trichotillomania or pica. Cross-sectional imaging and diagnostic endoscopy are complementary tools for diagnosis and surgical planning. Open excision with gastrotomy and enterotomy is safe and effective for large, multi-site trichobezoars not amenable to endoscopic removal. Multidisciplinary care that involves behavioral health specialists and a psychiatrist is essential to address the underlying compulsive behavior and reduce the risk of recurrence.
